# Aloesin Suppresses Cell Growth and Metastasis in Ovarian Cancer SKOV3 Cells through the Inhibition of the MAPK Signaling Pathway

**DOI:** 10.1155/2017/8158254

**Published:** 2017-06-18

**Authors:** Li-qian Zhang, Rong-wei Lv, Xiang-dong Qu, Xian-jun Chen, Hong-sheng Lu, Ying Wang

**Affiliations:** ^1^Gynecology Department, Taizhou Central Hospital, Zhejiang 318000, China; ^2^Clinical Laboratory, Taizhou Central Hospital, Zhejiang 318000, China; ^3^Pathology Department, Taizhou Central Hospital, Zhejiang 318000, China

## Abstract

Aloesin is an active constituent of the herb aloe vera and plays a crucial role in anti-inflammatory activity, ultraviolet protection, and antibacterium. We investigated the role and possible mechanisms of aloesin in the cell growth and metastasis of ovarian cancer. It was found that aloesin inhibited cell viability and cell clonality in a dose-dependent manner. It arrests the cell cycle at the S-phase and induced apoptosis in SKOV3 cells. In an in vivo experiment, it was observed that aloesin inhibited tumor growth. Moreover, it inhibited migration and invasion of cancer in SKOV3 cells. Interestingly, members from the mitogen-activated protein kinase (MAPK) signaling family became less phosphorylated as the aloesin dose increased. This suggests that aloesin exerts its anticancer effect through the MAPK signaling pathway. Our data also highlights the possibility of using aloesin as a novel therapeutic drug for ovarian cancer treatment.

## 1. Introduction

Ovarian cancer is one of the three common gynecological malignant tumors and ranks third in its rate of incidence. According to a recent statistic, there are 22,280 new cases of ovarian cancer per year in the United States, among which an estimated 15,500 patients die from this malignancy [1]. There are multiple factors which influence the development and progression of ovarian cancer; it is currently understood as a multistep disease that involves the coordinal interaction of multiple genes, and the accumulation of multiple molecular and morphologic changes within a cell. Surgery, chemotherapy, and radiotherapy are the three major therapeutic options for ovarian cancer. Unfortunately, prognosis is still poor due to limited therapeutic strategies, except for late diagnoses [2, 3]. Therefore, it is urgent to find a novel therapeutic treatment for ovarian cancer.

With a history of thousands of years of clinical practice, traditional Chinese medicine (TCM) plays an important role in maintaining the health of Asian peoples and is being increasingly applied all over the world. The aloe vera plant has a long history of use for medicinal purposes in China; currently, it is frequently used in herbal medicine for its anti-inflammatory activity, UV protection, antiarthritic properties, wound and burn healing capabilities, and antibacterial/anticancer properties [4–6]. There are several biologically active constituents in aloe vera, including aloe-emodin. Aloe-emodin has antiproliferative effects and induces cellular apoptosis [7–9]. It also produces anticancer activity in neuroectodermal tumors [10], nasopharyngeal carcinoma [11], lung squamous cell carcinoma [12], hepatoma cells [13], gastric cancer [14], and prostate cancer [15]. Aloe-emodin induces apoptotic cell death by oxidative stress and sustained c-Jun N-terminal kinase (JNK) activation [16]. Previous studies have demonstrated that aloe-emodin induces cell death through S-phase arrest in human tongue squamous cancer SCC-4 cells [17]. A previous study by the present authors also indicated that mTORC2 is a target of aloe-emodin, and aloe-emodin can strongly inhibit the AKT activation caused by PTEN loss [18].

Aloesin is another active constituent of aloe vera. Aloesin has been shown to be a potent and selective inhibitor of tyrosinase exhibited direct inhibitory effects on melanogenesis [18]. However, little is known about the role of aloesin in anticancer activity. All of the currently available literature has barely uncovered the signaling pathway that accounts for the anticancer activity of aloesin in human cancers. In this study, we evaluated the inhibitory effects of aloesin on the growth of various ovarian cancer lines. The results showed that aloesin kills ovarian cancer cells. We further show that aloesin arrests ovarian cancer cells at the S-phase of the cell cycle and induces apoptosis by inhibiting the activation of the MAPK signaling cascade. This leads to the inhibition of growth of cultured cells as well as the reduction of localized growth and dissemination of tumors in mice, showing promising preclinical activity of aloesin for ovarian cancer therapy.

## 2. Materials and Methods

### 2.1. Reagents and Cell Cultures

Aloesin was purchased from the National Institute for the Control of Pharmaceutical and Biological Products (Beijing, China), and the purity was at least 95% as determined by HPLC. Primary antibodies against MMP-9, MMP-2, and GAPHD were purchased from Abcam (Hong Kong, China). Antibodies against MEK, ERK, JNK, and p38 MAPK were obtained from Cellular Signaling Co. (NY, USA). The ovarian cancer cell lines OV-1063, CoC1, Cao V-3, OVCAR3, and SKOV3 were purchased from the American Type Culture Collection (ATCC, USA) and were maintained in Dulbecco's modified Eagle's medium (DMEM) (Invitrogen, CA, USA). The ovarian cancer cell lines were supplemented with 10% fetal bovine serum (FBS, Invitrogen) and 100 U/ml penicillin/streptomycin (Sigma, St. Louis, MO, USA). Cells were incubated in a humidified atmosphere at 37°C with 5% CO_2_. Cells were passaged every 2 d to obtain an exponential growth.

### 2.2. Western Blot Analysis

Total proteins were extracted using transfected cells. Extracted proteins were quantified using a BCA kit (Beyotime, Nantong, China). An equal amount of 50 ng proteins were loaded to a 12% SDS-PAGE gel and were then transferred onto PVDF membranes (pore size = 0.45 *μ*m) (Millipore, Billerica, MA, USA). After blocking in 5% skim milk in tris-based saline-tween 20 (TBST) for 60 min at room temperature, membranes were incubated with corresponding primary antibodies overnight at 4°C. The membranes were thereafter washed with TBST three times and further incubated with a secondary antibody for 1 h at room temperature. Enhanced chemiluminescence (ECL) solution was used to develop the immunoreactivity. GAPHD was synchronously detected as a loading control.

### 2.3. Cell Viability Assay and Colony Formation Assays

SKOV3 cells were seeded into 96-well plates at a density of 5 × 10^3^ cells/well, incubated overnight, and then treated with aloesin at different concentrations (0, 2.5, 5, 10, 20, and 40 *μ*M) for 48 h or incubated with 5 *μ*M of aloesin for 24, 48, and 72 h. Cell viability upon aloesin treatment was evaluated using the 3-[4,5-dimethylthiazol-2-yl]-2,5-diphenyl tetrazolium bromide assay (MTT, Sigma, St. Louis, MO, USA), as previously described [19]. To determine the long-term effect of aloesin on cell growth, colony formation assays were performed. Briefly, 500 cells were plated per well in 6-well plates. Cells were allowed to grow for 10 d, after which colonies were stained with crystal violet. The stained colonies were then photographed and manually counted.

### 2.4. Cell Cycle Analysis

SKOV3 cells were treated with different concentrations of aloesin (0, 2.5, 5, and 10 *μ*M) for 48 h. Then, SKOV3 cells were fixed with 80% cold ethanol and incubated with 0.5% Triton X-100 solution containing 1 mg/ml RNase A at 37°C for 30 m. Cell cycle was then detected by flow cytometry with PI staining according to a previous report [20].

### 2.5. Hoechst 33342 Staining

Apoptotic cells were identified on the basis of morphological changes in their nuclear assembly by observing chromatin condensation and fragment staining with Hoechst 33342. After treatment with different concentrations of aloesin for 48 h, the cells were washed in PBS and stained with 5 *μ*g/ml Hoechst 33342 reagent at 37°C in the dark for 10 min. Nuclear DNA staining was observed using a fluorescence microscope. In each group, five microscopic fields were randomly selected and counted.

### 2.6. Annexin V/PI Analysis

SKOV3 cells were treated with different concentrations of aloesin for 48 h. Apoptosis was analyzed using an Annexin V/PI apoptosis kit according to the manufacturer's instructions. The cells were harvested, washed twice with cold PBS, and resuspended at a density of 1 × 10^6^ cells/ml in 100 *μ*l of binding buffer containing 5 *μ*l of Annexin V-FITC and 1 *μ*l of PI working solution (100 *μ*g/ml). After incubation at room temperature in the dark for 30 min, the samples were analyzed by a flow cytometer (BD Biosciences).

### 2.7. Wound Healing Assay

SKOV3 cells were seeded into 6-well plates and treated with different concentrations of aloesin (0, 2.5, 5, and 10 *μ*M) for 24 h. Then, 10 *μ*l sterile pipette tips were used to create a vertical cross of constant width in the center of each well. After 24 h of growth, cells in the scraped wound were observed and photographed under a Nikon microscope at magnification of 200x for each group.

### 2.8. Transwell Migration and Invasion Assays

Transwell chambers with polycarbonate filters (8 *μ*m pore size) were purchased from Corning Co. (NY, USA). For the transwell migration assays, SKOV3 cells with the indicated doses of aloesin treatment were trypsinized and washed three times with FBS-free DMEM medium. Cells were then resuspended in FBS-free DMEM at a density of 2 × 10^5^ cells/ml and seeded into the upper chambers (100 *μ*l). For the lower chambers, 600 ml of DMEM medium containing 10% FBS was added. Cells were then allowed to migrate for 12 h, and membranes were thereafter stained with crystal violet. Cells that had transmigrated to the under surface of the filter were manually counted using a light microscope in five randomly selected fields. For invasion assays, the chambers were precoated with 50 *μ*l of Matrigel (1 : 30 dilution in a serum-free DMEM medium). Protocols were similar as described in the migration assays.

### 2.9. Mouse Xenograft and Metastasis Model

A xenograft model of ovarian cancer was established using SKOV3 cells. Briefly, 6-week-old athymic nude mice were randomly assigned to three groups: a control group (*n* = 5), aloesin-treated group (20 mg/kg, *n* = 5), and (40 mg/kg, *n* = 5). All mice were housed in specific pathogen-free (SPF) conditions according to the guidelines of the Ethics Committee of Taizhou Central Hospital. For each group of mice, SKOV3 cells (2 × 10^6^) were injected into the right flank. Mice from the experimental groups were injected with aloesin (20 mg/kg or 40 mg/kg) daily for three weeks. Mice were then monitored for the growth of tumors. Tumor length (L) and width (W) were measured twice a week. Tumor volume (TV) was calculated as TV = L × W^2^/2. Seven weeks after inoculation, all mice were sacrificed and the tumors were dissected. The dissected tumors were weighed and photographed. To induce lung metastasis, nude mice were injected with 1 × 10^6^ of SKOV3 cells by the lateral tail vein. After 1 month, the mice were euthanized and the lungs were removed for HE staining. All efforts were made to minimize suffering. Protocols for animal experiments were approved by the Ethics Committee from Taizhou Central Hospital.

### 2.10. Statistical Analysis

All data were expressed as mean ± standard deviation (SD). Differences between groups were assessed using the Student *t*-test. Differences with a *p* value less than 0.05 were considered statistically significant.

## 3. Results

### 3.1. Aloesin Inhibits Ovarian Cancer Cell Growth in a Dose- and Time-Dependent Manner

**Figure 1:**
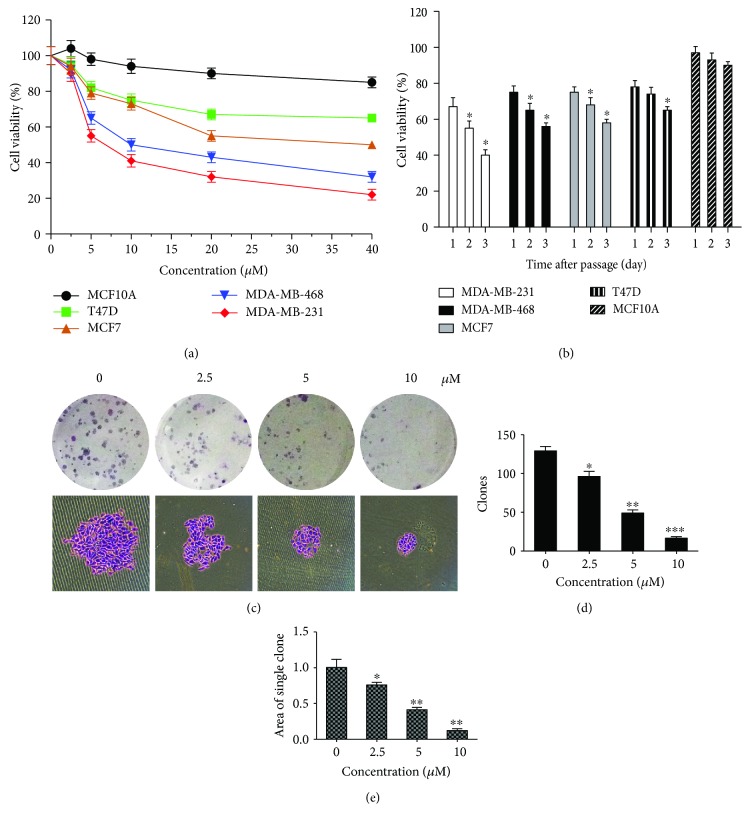
Aloesin inhibits SKOV3 cell growth in a dose- and time-dependent manner. (a) Five ovarian cancer cells were assayed for cell viability determination with the doses of aloesin treatment ranging from 2.5 *μ*M to 40 *μ*M. Cells with 0 *μ*M aloesin treatment were used as control. All groups of cells were cultured for 48 h. (b) At a fixed dose (5 *μ*M), aloesin inhibited five ovarian cancer cell viabilities in a time-dependent manner (24 h, 48 h, and 72 h). (c) Colony formation assays were performed using SKOV3 cells treated with indicated doses of aloesin. The clones were stained with crystal violet and shown. (d) In the colony formation assay, clones were formed and counted manually for each group of cells. (e) The size of each clone was also calculated and averaged. ^∗^*p* < 0.05; ^∗∗^*p* < 0.01; ^∗∗∗^*p* < 0.001.

To evaluate the toxic effect of aloesin ovarian cancer, five human ovarian cancer cell lines were treated with aloesin at different concentrations (0, 2.5, 5, 10, 20, and 40 *μ*M) and different times (24, 48, and 72 h). MTT assay showed that aloesin exhibited a concentration-dependent and time-dependent killing of diverse ovarian cancer cell lines (Figures 1(a) and 1(b)). The SKOV3 cell line was more sensitive to aloesin than other cell lines. Aloesin had a significantly potent toxic effect with an IC50 value of around 5 *μ*M in SKOV3 cell lines. Therefore, SKOV3 cell lines were chosen as the optimal cell models for subsequent functional analyses. SKOV3 cell line was treated with aloesin in dosages ranging from 2.5 to 10 *μ*M in vitro. We adopted the doses 0, 2.5, 5, and 10 *μ*M of aloesin and performed colony formation assays. It was visually observed that colonies were smaller in size with increasing doses of aloesin (Figure 1(c)). Quantitative analysis further revealed that colony numbers also decreased with increasing doses of aloesin (Figure 1(d)). Consistently, the average area of a single clone was also decreasingly smaller as the aloesin dose increased (Figure 1(e)). These data suggest that aloesin inhibits ovarian cancer cells growth in a dose- and time-dependent manner.

### 3.2. Aloesin Arrests the Cell Cycle and Promotes Cell Apoptosis in SKOV3 Cells

**Figure 2:**
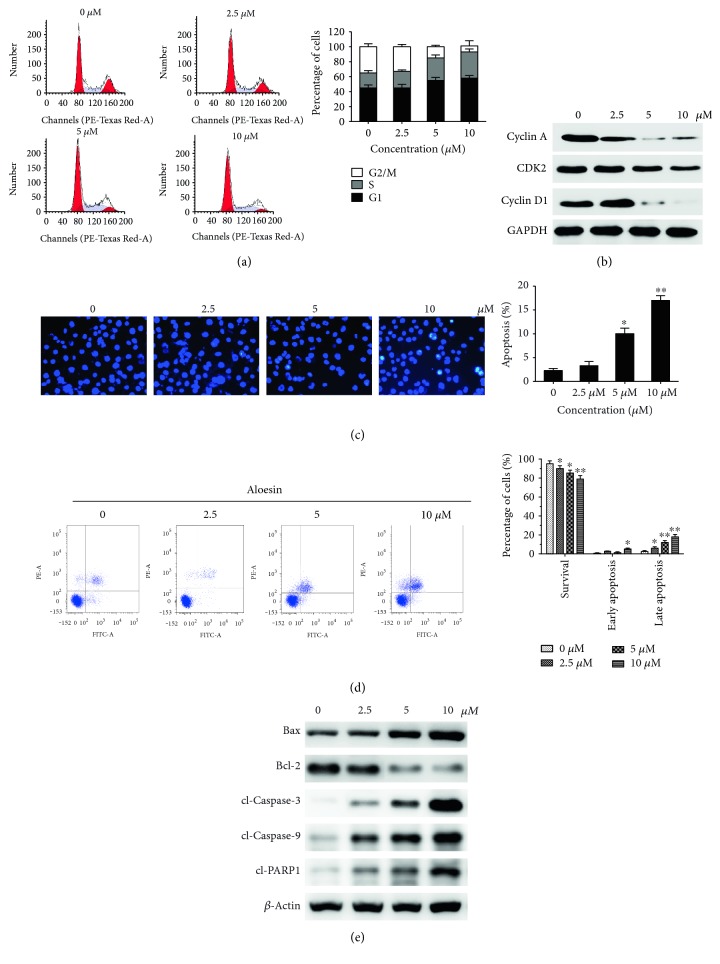
Aloesin arrests cell cycle and promotes cell apoptosis in SKOV3 cells. (a) SKOV3 cells without or with different doses of aloesin treatments were subject to cell cycle analysis by flow cytometry. Horizontal ordinate: channels (PE-A). Ordinate: cell number. (b) Western blot analysis of cell cycle-related proteins in both cell lines. *β*-actin was used as a loading control. Data represent the mean ± SD of three independent experiments. (c) Changes in apoptotic nuclear morphology were observed by Hoechst 33342 staining and visualized by fluorescent microscopy. (d) Cells were analyzed by flow cytometry with Annexin V-FITC/PI staining after aloesin treatment. Annexin V versus PI plots from the gated cells showed the populations corresponding to viable (Annexin V−/PI−) and necrotic (Annexin V−/PI+), early (Annexin V+/PI−), and late (Annexin V+/PI+) apoptotic cells. Horizontal ordinate: Annexin V-FITC. Ordinate: PI-PE. (e) Western blot analysis of apoptosis-related proteins in both cell lines. *β*-actin was used as a loading control. Data represent the mean ± SD of three independent experiments. ^∗^*P* < 0.05, ^∗∗^*P* < 0.01 versus control.

We further examined the mechanism behind how aloesin impairs viability in the ovarian cancer cell line. Cell cycle progression was assessed. It was shown that the cell cycle was arrested when SKOV3 cells were treated with aloesin. More importantly, the percentage of cells in the G2/M phase dropped gradually with increasing doses of aloesin. In contrast, the percentages of cells in the S-phase increased in aloesin-treated SKOV3 cells (Figure 2(a)). These results indicate that aloesin could arrest the cell cycle at S-phase in a dose-dependent manner. Western blot further confirmed that the levels of the S-G2/M-related proteins cyclin A, CDK2, and cyclin D1 were downregulated in the SKOV3 cell line following aloesin treatment (Figure 2(b)).

**Figure 3:**
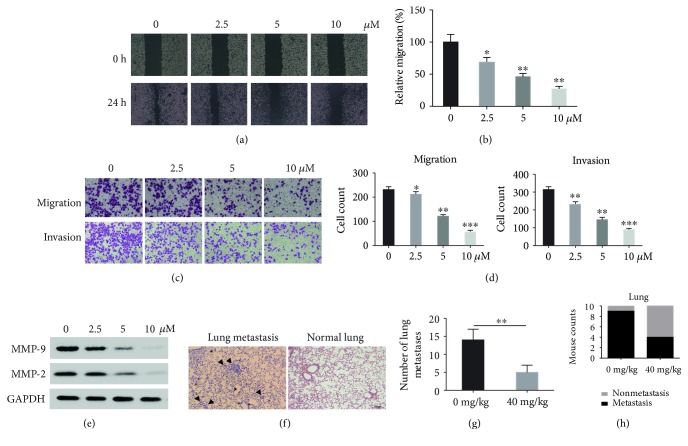
Aloesin inhibits cell migration and invasion in SKOV3 cells. (a) Wound healing assay was performed to observe the effects of aloesin treatment on SKOV3 cell wound recovery ability. (b) Quantification of the healed wound area. (c) Transwell migration and invasion assays were performed to observe the effects of aloesin treatment on SKOV3 cell migration and invasion abilities. For each group of cells, five fields were randomly photographed and representative images were shown here. (d) In the transwell migration and invasion assays, transmigrated cells were counted from the photographed 5 fields in each group. The average cell numbers were calculated in each group. (e) Western blot analysis of major matrix metalloproteinase MMP-2 and MMP-9 expression in SKOV3 cells with distinct doses of aloesin. ^∗^*p* < 0.05; ^∗∗^*p* < 0.01; ^∗∗∗^*p* < 0.001. (f) Representative photos of histological H&E staining of lung metastasis were shown for each group. (g) A bar graph summarizes the number of lung metastasis node in the two groups. (h) A bar graph summarizes the incidence of lung metastasis in the two groups.

Next, Hoechst 33342 staining was performed to examine the nuclear morphology. In control cells, the nuclei were stained weakly and homogeneously blue, whereas in cells treated with aloesin, some bright chromatin condensation and nuclear fragmentation was observed (Figure 2(c)). The numbers of apoptotic nuclei containing condensed chromatin increased significantly as the aloesin concentration increased. Based on these data, aloesin appeared to cause apoptosis in SKOV3 cell line. Apoptosis was confirmed by flow cytometry analysis with Annexin V-FITC/PI staining. As shown in Figure 3(d), a marked dose-dependent increase in late stages of apoptosis was observed in the SKOV3 cell line following aloesin treatment. Apoptosis is a type of programmed cell death that is caspase dependent. When SKOV3 cells were treated with aloesin at different concentrations, significant proteolytic cleavage of caspase-3, caspase-9, and PARP1 was detected using western blot (Figure 2(e)). The levels of pro- and antiapoptotic mitochondria proteins Bax and Bcl-2 were also visualized by western blot. Aloesin increased the expression of Bax and conversely decreased Bcl-2 in SKOV3 cell line. The results of these different apoptosis assays reveal significant features of apoptosis, which strongly suggest that aloesin-mediated inhibition of cell growth in ovarian cancer cell lines is closely correlated with enhanced apoptosis.

### 3.3. Aloesin Inhibits Tumor Growth in a Xenograft Model of Ovarian Cancer

**Figure 4:**
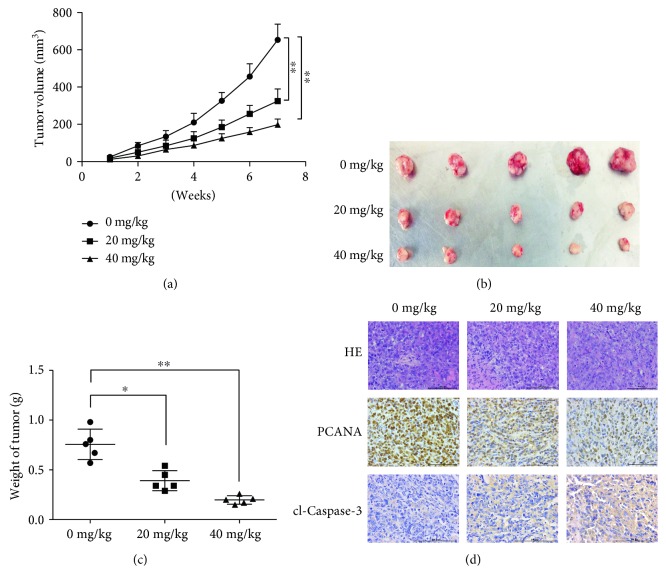
Aloesin inhibits tumor growth in a xenograft model of ovarian cancer. (a) A xenograft model of ovarian cancer was established using SKOV3 cells. Mice from each group were injected with aloesin (20 mg/kg or 40 mg/kg) daily until 7 weeks. Mice without aloesin injection was fed as control. Tumor volumes in each group were calculated and shown. (b) Seven weeks after inoculation, tumors from each group of mice were dissected. (c) Dissected tumors were weighed. The average weight of tumors from each group of mice was calculated. (d) The expression of PCNA and cleaved caspase-3 was detected in xenografts by IHC. ^∗^*p* < 0.05; ^∗∗^*p* < 0.01.

We established a xenograft model of ovarian cancer using SKOV3 cells. Experimental groups were injected with 20 mg/kg or 40 mg/kg of aloesin daily for seven weeks. Tumor size was periodically monitored. The tumor volumes became different between the experimental and control groups after four weeks, and the differences were significantly large at seven weeks. The tumor volumes of the control group were significantly larger than those of the experimental groups (Figures 4(a) and 4(b)). The average weights of dissected tumors were also significantly different among the experimental and control groups; tumors were largest in the control group and smaller in the experimental groups (Figure 4(b)). Moreover, in aloesin-treated xenograft tumors, fewer proliferative cells and more apoptotic cells were observed by detecting proliferating cell nuclear antigen (PCNA) and cleaved caspase-3, respectively (Figure 4(d)). These data suggest aloesin could inhibit ovarian cancer growth in vivo.

### 3.4. Aloesin Inhibits Cell Migration and Invasion in SKOV3 Cells

The effects of aloesin on SKOV3 cell metastasis were subsequently assessed. As shown in Figure 3(a), higher doses of aloesin inhibited the wound closure process compared to control cells; approximately 70% of wound closure was inhibited in the 10 *μ*M concentration of aloesin (Figure 3(b)). In the transwell assays, migrated cells and invaded cells were visually inhibited after the treatment of aloesin (Figure 3(c)). Most control cells migrated to the lower surface, whereas 80% of cells did so after 2.5 *μ*M of aloesin treatment, and only 20% of cells successfully migrated when the dose of aloesin was increased to 10 *μ*M (Figure 3(d), left panel). For the invasion assay, 70% of cells did that after 2.5 *μ*M of aloesin treatment and only 25% of cells successfully invaded when the dose of aloesin was increased to 10 *μ*M (Figure 3(d), right panel). Besides, MMP-2 and MMP-9 were dose dependently decreased by aloesin treatment (Figure 3(e)). In order to assess the contribution of aloesin to tumor metastasis in vivo, we employed the animal model of experimental pulmonary metastasis. We inoculated the SKOV3 cells through intravenous injections in the tails of immunodeficient nude mice. The numbers of metastatic colonies in the lungs were significantly less in mice treated with aloesin than in those treated with PBS (Figures 3(f) and 3(g)). Furthermore, there was a higher incidence of tumor nodules in the lungs of mice without aloesin treatment compared to those mice treated with aloesin (Figure 3(h)). Our data suggests that aloesin inhibits the metastasis of ovarian cancer cells in vivo and in vitro.

### 3.5. Aloesin Inhibits the Phosphorylation of the MAPK Signaling Pathway

**Figure 5:**
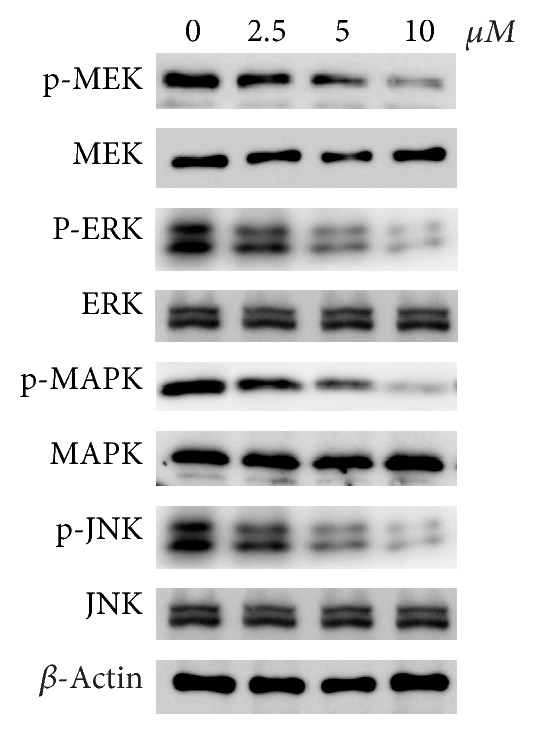
Aloesin inhibits the phosphorylated levels of MAPK signaling pathway. Western blot analysis of the protein levels of MAPK family members in SKOV3 cells with distinct doses of aloesin treatment. Both the total level and phosphorylated level of each member were detected. *β*-actin was developed as a loading control.

Western blot analysis showed that after treatment of aloesin in SKOV3 cells, the MAPK family members, which are serine-threonine kinases that regulate a wide variety of cellular functions. Although the total protein levels of MEK, ERK, MAPK, and JNK remained unchanged, the phosphorylated levels of these four proteins were significantly decreased. This result indicates that the MAPK signaling pathway was inactivated by aloesin treatment (Figure 5).

## 4. Discussion

Ovarian cancer remains a great threat for female health around the world; it is estimated that only approximately 30% of patients have a 5-year survival rate [21]. Aloesin is the active constituent of the Chinese medicinal herb aloe vera and has been shown to exert anti-inflammatory effects, UV protection, and antibacterial properties [4–6]. The present study investigated the possible therapeutic values of aloesin against ovarian cancer and its potential pathways.

Aloesin exposure resulted in a dose- and time-dependent decrease in the viability of all tested ovarian cell lines. In a colony assay, the clonogenic potential of SKOV3 cells were inhibited by aloesin in a dose-dependent manner. The cell cycle was arrested when SKOV3 cells were treated with aloesin, and the G2/M phase dropped quickly with increasing aloesin concentrations. A marked dose-dependent increase in apoptosis, especially late apoptosis, was observed in SKOV3 cell line following aloesin treatment. Furthermore, aloesin potently inhibited tumor growth in vivo. In all, our data indicated that aloesin has a potential anticancer effect in ovarian cancer. We also investigated the migration and invasion of SKOV3 cells treated with aloesin; these cells were less able to migrate and invade. MMP-2 and MMP-9, which are markers for distant metastasis, were also decreased in the aloesin treatment groups, which was further supportive of aloesin inhibition. Collectively, our data show that aloesin has the potential to inhibit cell growth and metastasis in ovarian cancer.

In addition, our data showed that the phosphorylation levels of the MAPK signaling pathway (p-ERK, p-JNK, and p-p38 MAPK) were decreased by aloesin treatment in SKOV3 cells, showing that aloesin might regulate the MAPK signaling pathway in ovarian cancer. The mammalian MAPK family consists of ERK, p38, and JNK, and each member has several isoforms: ERK1 to ERK8; p38*α* (MAPK14), p38*β* (MAPK11), p38*γ* (MAPK12), and p38*δ* (MAPK13); and JNK1 (MAPK8), JNK2 (MAPK9), and JNK3 (MAPK10). Each MAPK signaling cascade consists of at least three layers: a MAPK kinase (also known as RAF), a MAPK kinase (also known as MEK), and a MAPK kinase. Activated MAPKs phosphorylate numerous substrates and thereby regulate many important cellular processes such as differentiation, proliferation, survival, and cell adhesion. In this study, it was observed that aloesin dose dependently decreased the phosphorylation levels of ERK, JNK, and p38 MAPK as well as MEK, suggesting that aloesin regulates the MAPK signaling pathway. However, a previous report has found that only p38 and JNK/MAPK were involved in aloe vera-mediated cell apoptosis in leukemia U937 cells. ERK was intriguingly not associated with the above activity, which contradicts our data. We speculated that this contradiction might be due to the different cancer types and, if this was true, distinct mechanisms might underlie the aloesin-mediated anticancer activity in different cancer types. However, more work needs to be done.

In summary, the present study identified aloesin as a novel therapeutic compound to inhibit tumor growth and metastasis in ovarian cancer. The anticancer activity of aloesin may be through the MAPK signaling pathway. Our data highlights the possibility of using aloesin as a novel therapeutic drug for ovarian cancer treatment.
